# Research on Fatigue Driving Detection Technology Based on CA-ACGAN

**DOI:** 10.3390/brainsci14050436

**Published:** 2024-04-27

**Authors:** Han Ye, Ming Chen, Guofu Feng

**Affiliations:** College of Information, Shanghai Ocean University, No. 999 Huchenghuan Road, Shanghai 201306, China

**Keywords:** EEG, fatigue driving detection, conditional generative adversarial network, attention mechanism, bottleneck residual

## Abstract

Driver fatigue represents a significant peril to global traffic safety, necessitating the advancement of potent fatigue monitoring methodologies to bolster road safety. This research introduces a conditional generative adversarial network with a classification head that integrates convolutional and attention mechanisms (CA-ACGAN) designed for the precise identification of fatigue driving states through the analysis of electroencephalography (EEG) signals. First, this study constructed a 4D feature data model capable of mirroring drivers’ fatigue state, meticulously analyzing the EEG signals’ frequency, spatial, and temporal dimensions. Following this, we present the CA-ACGAN framework, a novel integration of attention schemes, the bottleneck residual block, and the Transformer element. This integration was designed to refine the processing of EEG signals significantly. In utilizing a conditional generative adversarial network equipped with a classification header, the framework aims to distinguish fatigue states effectively. Moreover, it addresses the scarcity of authentic data through the generation of superior-quality synthetic data. Empirical outcomes illustrate that the CA-ACGAN model surpasses various extant methods in the fatigue detection endeavor on the SEED-VIG public dataset. Moreover, juxtaposed with leading-edge GAN models, our model exhibits an efficacy in in producing high-quality data that is clearly superior. This investigation confirms the CA-ACGAN model’s utility in fatigue driving identification and suggests fresh perspectives for deep learning applications in time series data generation and processing.

## 1. Introduction

Globally, the issue of road safety remains a pressing concern, with the Global Health Authority reporting that traffic-related fatalities annually claim the lives of around 1.25 million people worldwide [1], establishing these accidents as the leading death cause among young people aged 15 to 29 years [2].

Driving fatigue significantly compromises driving safety and further exacerbates the problem. Research conducted in Europe revealed that around 17% of drivers have experienced drowsiness while operating vehicles, with 7% reporting involvement in accidents due to this condition [3]. Consequently, the development of advanced fatigue monitoring methodologies is imperative for enhancing road safety.

Current strategies for monitoring driver fatigue predominantly examine three aspects: vehicle dynamics [4], drivers’ behavioral patterns [5,6], and physiological signals [7]. These methods assess drivers’ attention levels by evaluating operational data from the vehicle and observing drivers’ behavioral changes, including facial expressions and eye movements. Nevertheless, these approaches encounter obstacles such as privacy concerns and the influence of environmental variables. Fatigue detection techniques based on physiological measurements, like the electroencephalogram (EEG), electrocardiogram (ECG), and electrooculogram (EOG), offer objective insights into a driver’s fatigue levels. In particular, different wave rhythms in EEG, such as δ, θ, α, β, and γ waves, can reflect the driver’s mental state and physiological activities [8], which are useful for distinguishing between fatigue and wakefulness, and are the key to fatigue detection. Despite the non-invasive nature and high accuracy of EEG-based methods, challenges persist in signal processing, limited sample sizes, and unbalanced data distribution. First, EEG recordings often encounter interference from external environmental factors and internal physiological activities, potentially compromising the integrity of the signal and complicating the subsequent data analysis processes. Second, the collection and labeling of EEG data requires specialized knowledge, so there is the problem of a limited sample size, which limits the depth and breadth of the research. In addition, when dealing with multi-category medical datasets, an unbalanced data distribution often leads to an uneven classification performance of the model, which is mainly due to the natural differences in the frequency of different events.

To enhance the accuracy of EEG signal decoding, it is imperative to holistically optimize the feature extraction and classification methodologies while pursuing more effective algorithms. EEG signals are imbued with abundant frequency, spatial, and temporal information, pivotal for elucidating the brain’s activity patterns. Nonetheless, conventional studies on EEG signals predominantly focus on a limited scope, analyzing merely one or two dimensions of information. For a spatial analysis, this is accomplished by analyzing the differences in activity recorded by electrodes in different regions of the brain, which can be accomplished in a variety of ways, such as converting EEG signals into topological maps [9], exploring spatial correlations between electrodes using a Recurrent Neural Network (RNN), or constructing 2D representations of electrode data for deep learning model training [10]. Conversely, temporal analysis accentuates the dynamics of brain activity over time, employing Convolutional Neural Networks (CNNs) and Long Short-Term Memory (LSTM) networks to adeptly extract EEG signal time series features [11]. Investigating novel architectures for the efficient extraction of EEG signals’ multidimensional features warrants further exploration.

The efficacy of deep learning models significantly depends on extensive training data. In scenarios with limited samples, data augmentation techniques can substantially enhance the models’ decoding performance. In 2014, a team led by Ian Goodfellow unveiled a novel framework for deep learning, known as the generative adversarial network (GAN) [12], that addresses data scarcity by synthesizing new, unseen data. Traditional GANs, however, grapple with specifying the generated data’s type and quality. Conditional generative adversarial networks (CGANs) [13] address this problem by adding additional conditional information, such as category labels or partial image information, to the generators and discriminators, improving the controllability and targeting of data generation. Nevertheless, CGANs still face challenges when dealing with time series data with multiple signal categories, such as each category needing to be trained separately, which is a time-consuming and inefficient process. In addition, the available datasets are usually small, and the distribution of data categories is not balanced, which makes the model prone to overfitting when training on categories with low amounts of data. Data shortage and an uneven category distribution are challenges when training multiple GAN models.

To address the data imbalance problem, researchers have adopted various strategies to mitigate its negative impact on model performance, including undersampling the overrepresented classes, oversampling the underrepresented classes, giving higher weights to the underrepresented classes, and using improved evaluation metrics [14]. While these approaches partially alleviate the issue of imbalanced datasets, they can lead to the disappearance of essential data, negatively affecting the model’s capacity to assimilate knowledge.

Recent endeavors have explored the adoption of RNN-based GAN architectures for synthesizing time series data, exemplified by C-RNN-GAN [15] and SigCWGAN [16]. Notwithstanding, RNNs struggle with the ability to connect more distant time steps in long sequences. Conversely, the Transformer architecture has emerged as a groundbreaking development in neural network technologies, outperforming conventional models across various fields, such as image classification and sequence data processing [17,18]. The Transformer’s innovative design and its proficient mitigation of the gradient vanishing dilemma in lengthy data sequences underscore its efficacy. This framework has been applied successfully in a variety of fields, such as natural language processing and computer vision, with recent studies investigating its integration with GAN architectures to enhance data generation quality and optimize the training process [19].

This research endeavored to enhance the production of sequential data and augment the precision of fatigue driving detection through deep learning methodologies. The contributions are delineated as follows:

1. We developed an innovative conditional generative adversarial network that leverages convolution and attention mechanisms (CA-ACGAN). Our empirical analysis confirmed that the CA-ACGAN model surpasses several established methods in fatigue detection tasks, utilizing the SEED-VIG public dataset for evaluation.

2. The CA-ACGAN was also designed to conditionally generate multi-class time series data with labels. The superiority of our model in generating data quality was illustrated by contrasting qualitative metrics with those of other leading-edge GAN models. Moreover, we adopted a classification confidence-based selection technique to sift through and retain high-quality generated data. This approach facilitates an effective amalgamation of authentic and artificially generated data, thereby augmenting the classification accuracy of the model.

3. The CA-ACGAN amalgamates the strengths of CNNs and Transformes, thereby enhancing its capability for feature extraction. In combining a four-dimensional feature mapping framework, the attention module and the bottleneck residual module, the model’s ability to recognize the essential frequency and spatial domains during fatigue analysis is improved. Furthermore, the introduction of the Transformer encoder’s self-attention mechanism optimizes feature representation and diminishes feature redundancy. This optimization significantly bolsters the model’s efficacy in extracting temporal features.

## 2. Method

The following segment outlines the methodology for transforming raw EEG signals into four-dimensional (4D) feature data, as well as the overall structures of the CA-ACGAN model and its constituent modules.The CA-ACGAN model is capable of accurately recognizing and generating high-quality 4D feature data.

This paper’s key symbols and terms are compiled in Abbreviations.

### 2.1. Model Structure

**Figure 1:**
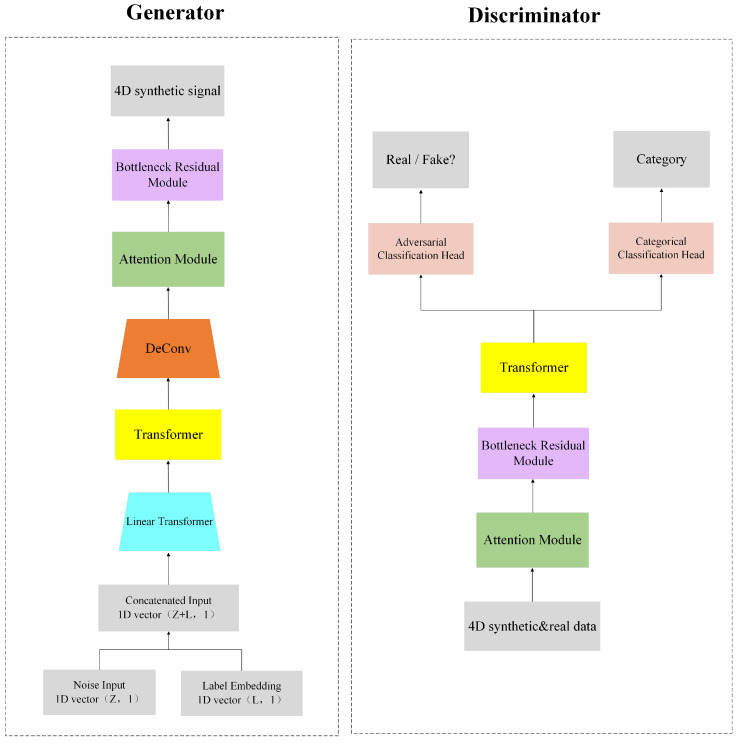
Overall framework of CA-ACGAN model.

Figure 1 illustrates the overall framework of the CA-ACGAN model, which comprises a pair of key units: a generator and a discriminator. Three main modules are embedded in the ACGAN (a GAN with classification header) model: an attention module, a bottleneck residual module, and the Transformer module, which together optimize the system’s capacity for data analysis. With the attention module, the model’s frequency and spatial feature recognition is enhanced; the bottleneck residual module optimizes the computational process to extract frequency and spatial features; and the Transformer module assists the model to understand the long-term dependencies among the data.

In terms of specialized functionality, the generator *G* transforms the concatenated vector, comprising the noise vector *z* and the target categorical label *l*, into data $W_{n}$ through a linear mapping. The randomization in the production of the label *l* enhances the generator’s capability to produce data across various categories.The Transformer module receives $W_{n}$ as its input. In the data processing phase, the generator employs a Deconvolution (DeConv) layer and a reshape operation to expand the data $W_{n}^{\prime }$, which has been processed by the Transformer module, into 4D feature data $T_{n}$. This serves as the input for the attention module and the bottleneck residual module, which facilitates the generation of synthetic signal $Y_{n}$ characterized by a 4D structure, denoted as $G\left(z,l\right)\to Y_{n}$. It is noteworthy that $W_{n}$, $W_{n}^{\prime }$, $T_{n}$, and $Y_{n}$ are instantiated within specific dimensional spaces, denoted as $W_{n},W_{n}^{\prime }\in R^{64\times 2T}$ and $T_{n},Y_{n}\in R^{h\times w\times d\times 2T}$, thereby delineating the structural framework of the generated data. In the document presented, it has been established that the value of 2T equals 16, with the parameters h, w, and d assigned the values of 6, 9, and 5 respectively.

The objective of discriminator *D* is to assess whether the input signal is authentic and to identify its respective category. Specifically, the real EEG data needs to be converted into 4D feature data $X_{n}$, and either $X_{n}$ or the generated 4D data $Y_{n}$ can be used as input for the discriminator. The data processing procedure encompasses a feature extraction phase, which also involves the deployment of the attention module, the bottleneck residual module, and the Transformer module. The conditional discriminator is equipped with two classification heads, one head is responsible for recognizing the truth of the signal ($D_{\mathrm{adv}}$), and the other head is used to identify the category of the signal ($D_{\mathrm{cls}}$), i.e., $X_{n}\to \left\{D_{\mathrm{adv}}\left(X_{n}\right),D_{\mathrm{cls}}\left(X_{n}\right)\right\}$ and $Y_{n}\to \left\{D_{\mathrm{adv}}\left(Y_{n}\right),D_{\mathrm{cls}}\left(Y_{n}\right)\right\}$. Such a configuration not only improves the accuracy of the model in recognizing the authenticity of data, but also enhances its classification performance, which provides effective support for handling complex data situations.

#### 2.1.1. Constructing EEG Data with 4D Features

In recent explorations, there has been a growing trend toward amalgamating the dimensions of frequency, spatial orientation, and time within EEG data into a unified four-dimensional feature construct. This advancement is poised to enhance methodologies in fields like motor imagery processing and the identification of emotional states. A novel method for EEG-based emotion recognition using a four-dimensional convolutional recurrent neural network (4D-CRNN) [20], which improves accuracy by integrating the frequency, spatial, and temporal information of multichannel EEG signals, has been proposed. Concurrently, the 4D Attention-Based Neural Network (4D-aNN) represents an innovative approach developed to facilitate the recognition of emotions from EEG signals [21]. Some approaches to motor imagery processing also offer novel insights into the precise parsing of brain activity by incorporating the multidimensional aspects of EEG signals [22].

**Figure 2:**
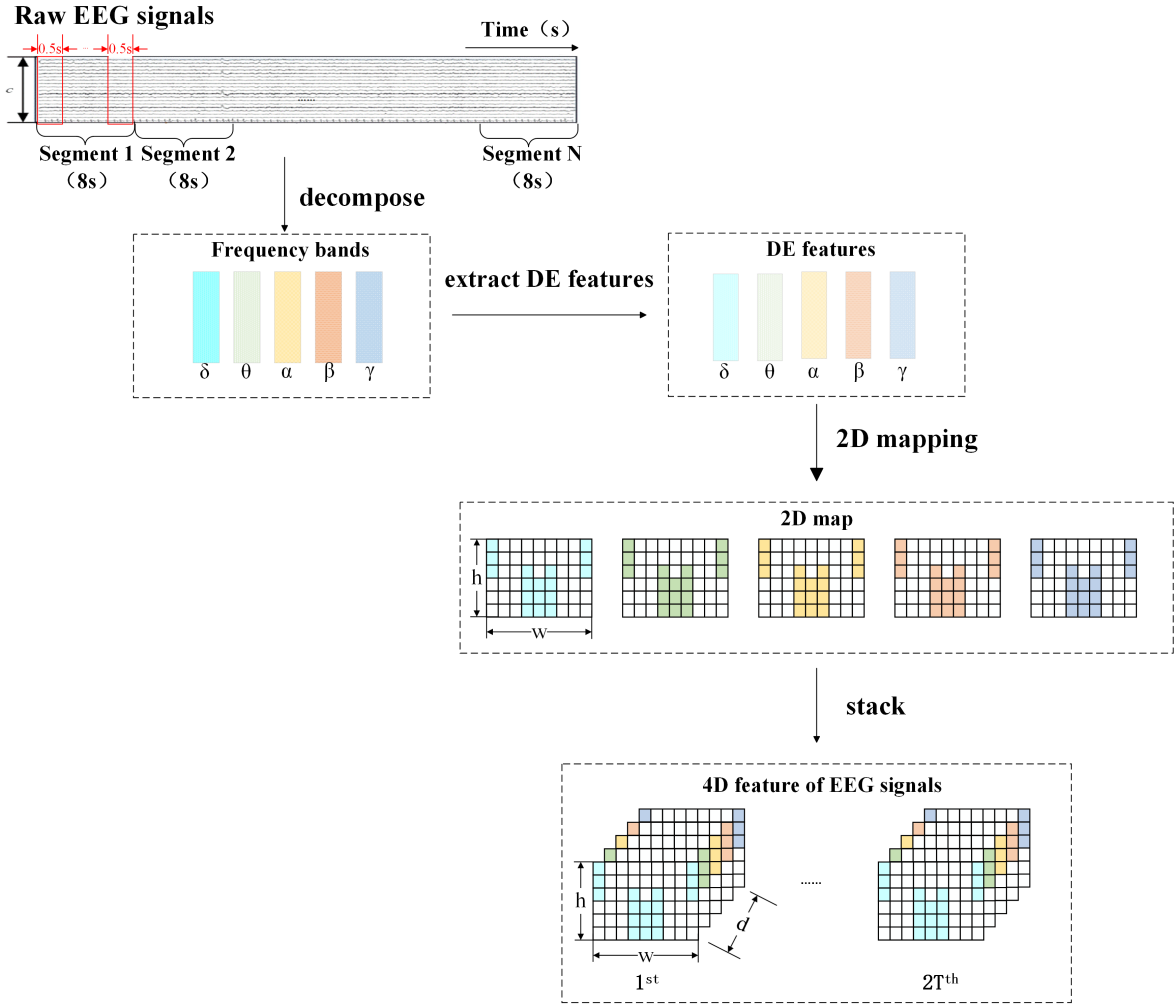
4D feature construction process.

In referring to the extant literature, the conversion of raw EEG data into a 4D format encompasses several critical steps, as depicted in Figure 2. Initially, raw EEG signals undergo preliminary preprocessing operations, including amplification and filtering, aimed at eliminating background noise and accentuating useful signals. Subsequently, the preprocessed signals are segmented into multiple temporal segments tailored to the experimental design and analytical requirements. Afterward, various feature extraction techniques are employed in order to identify characteristics of EEG signal frequency ranges across different time intervals. This step generates a three-dimensional data structure incorporating both frequency and spatial (i.e., electrode position) dimensions. In the final stage, to accommodate the needs of further analysis or model training, these 3D data are augmented into a 4D structure through techniques such as stacking, with the fourth dimension embodying the temporal aspect. Through this series of processing, the raw EEG signals are transformed into 4D data that can comprehensively reflect the dynamics of brain activity and its characteristics.

Next, we elucidate the construction process in detail. Initially, we introduce a novel feature termed differential entropy (DE) [23], which demonstrates stability and efficacy as a characteristic in classifying fatigue. This feature is employed to quantify the complexity inherent in continuous random variables. Calculating differential entropy relies on the random variable’s probability density function, as demonstrated in Equation (1):

(1) DE=−∫f(x)log(f(x))dx

In this context, *x* represents a random variable, whereas f(x) signifies its corresponding probability density function.

A SEED-VIG dataset’s raw EEG signal is represented as $S_{n}$, $S_{n}\in R^{m\times rT}$, where *m* denotes the quantity of electrode channels available, and *r* signifies the rate of sampling. Specifically, *m* is assigned a value of 17, and *r*, a value of 200. To align each EEG datum with its respective label, we segmented the raw EEG signals into multiple non-overlapping segments, each 8 s in length. This segmentation approach leverages the dataset’s methodology of computing a label every 8 s, which is then assigned to the corresponding EEG segment. To concurrently augment the dataset, the segments derived from EEG signals were subsequently segmented into time frames, amounting to 2T in total, with each frame spanning a duration of half a second. EEG can be decomposed into five distinct frequency bands: δ (1–4 Hz), θ (4–8 Hz), α (8–14 Hz), β (14–31 Hz), and γ (31–51 Hz), which have been strongly associated with fatigue levels in humans. It has been found that analyzing information from these frequency bands in combination provides a more accurate monitoring of fatigue status than relying on a single band alone. Hence, we processed the EEG signals by extracting these five standard frequency bands utilizing a Butterworth filter and computing their differential entropy (DE). This procedure transformed the original EEG signal segments into DE segments, denoted as $D_{n}\in R^{m\times d\times 2T}$, where *d* signifies the count of distinct frequency bands (d=5).

**Figure 3:**
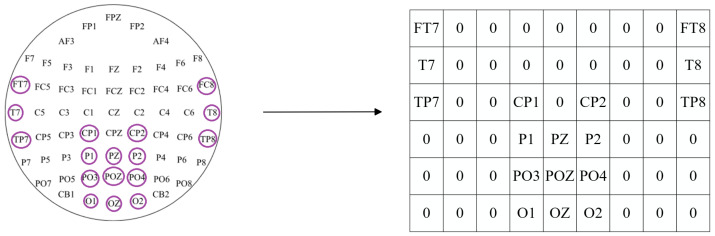
2D mapping.

The preceding process fails to maintain the spatial relationships inherent to electrode placements. To address this limitation, DE features are transformed into a localized 2D mapping, informed by the electrodes’ physical arrangement as illustrated in Figure 3. This can better represent the spatial relationship between electrodes. With this mapping, we generate a minimal 2D matrix that covers all the electrode locations with significant effects, and the unused electrode locations are filled with 0. Through the 2D matrix, we synthesize to reveal 3D characteristics. Subsequently, these temporal 3D characteristics, $D_{n}$, are superimposed to form 4D attributes, $X_{n}$, which can be represented as $X_{n}\in R^{h\times w\times d\times 2T}$. Here, *h* denotes the vertical measurement, and *w* signifies the horizontal dimension of the 2D diagram. The dataset’s spatial arrangement, consisting of 17 channels, manifests as a 2D diagram with vertical and horizontal dimensions measuring 6 and 9 units, respectively.

To encapsulate, the four-dimensional feature $X_{n}$ encompasses not only the distinct entropy traits observed in EEG signals within five distinct frequency domains, but also encapsulates spatial information pertaining to the electrode placements while maintaining the temporal continuity among successive time windows. The discriminator *D* receives the genuine EEG signals from the 4D characteristics as its input, with the purpose of allowing the discriminator to learn its features in order to differentiate between the genuine signals and the synthetic signals.

#### 2.1.2. Auxiliary Classifier Generation Adversarial Network (ACGAN)

**Figure 4:**
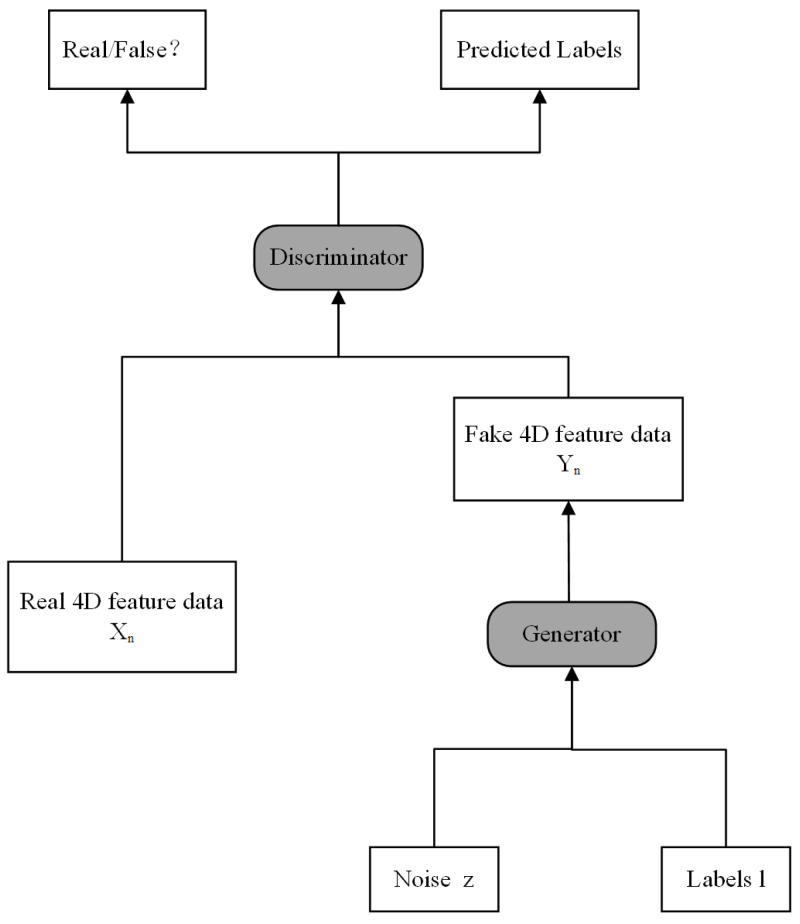
ACGAN structure.

The practice of embedding labels on generators and discriminators fails to generate meaningful synthetic data. In response, we implemented a methodology that leverages label information as a conditional input for the generator. This adjustment not only streamlines the generation of multi-category data, but also enhances the overall quality and diversity of the output. In addition, a categorization header is introduced as part of the assisted categorization task, which is capable of extracting information from the middle layer of the discriminator to predict the data’s category. The classification outcome for fatigue detection, associated with the initial EEG signal fragment, is determined after contrasting the prediction with the classification threshold, as depicted in Figure 4.

#### 2.1.3. Spatial and Frequency Attention

Roy introduced a novel method aimed at enhancing the efficacy of image segmentation tasks. This method involves the parallel integration of spatial and channel Squeeze and Excitation (SE) modules within a fully convolutional network framework [24]. The investigation delineates three distinct variants of the SE module: cSE, sSE, and scSE. The cSE variant amplifies the channel dimension, the sSE variant targets the spatial dimension, while the scSE variant concurrently enhances both the channel and spatial dimensions. Empirical outcomes indicate that the incorporation of the SE module markedly enhances the precision of image segmentation tasks without contributing to increased model complexity. These findings usher in novel avenues for research and furnish the medical field with innovative technological instruments, underscoring the SE modules’ capacity to significantly boost model performance.

**Figure 5:**
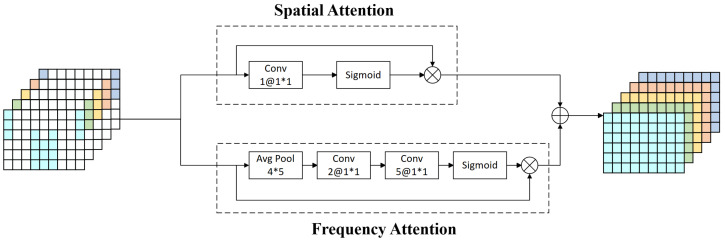
The attention module in the discriminator, which has the same structure as the module in the generator.

In this study, we introduced an attention module comprising two distinct components: spatial attention and frequency attention. It incorporates mechanisms for focusing on specific spatial locations and frequency ranges. The architecture of the attention module is identically implemented within the generator and the discriminator, as shown in Figure 5.

Initially, the framework for spatial attention is engineered to leverage the 2D layout information of the electrode placements, thereby assigning specific weights to each temporal segment. This process generates a new spatial attention feature map using a 1×1×1 convolutional kernel for feature compression in the frequency dimension and then applying a Sigmoid function activation. This step highlights the key features in the spatial dimension and merges them with the original features to update the time segments at the spatial level.

Subsequently, the frequency attention mechanism is tasked with examining the interactions across different frequency bands (totalling five) within each temporal slice to allocate corresponding weights. By reducing the dimensions of the original feature map from a structure of 6×9×5 down to a compact format of 1×1×5, and applying dual convolutional layers for the further processing of the feature matrix, followed by Sigmoid function activation, a novel map focusing on frequency-based attention features was produced. This method prioritizes the relative significance among the five frequency ranges, merging them with the primary characteristics to update the temporal segments in the frequency dimension.

By integrating spatial and frequency attention-weighted time segments, we fine-tuned the spatial versus frequency dimensions of each time segment. This approach effectively enhances the model’s attention to spatially salient features and frequency-critical features, while suppressing minor features, thus significantly enhancing the comprehensive efficacy of the framework.

Within the CA-ACGAN architecture, the attention module is pivotal, orchestrating the intricate amalgamation of 4D features across the spatial and frequency domains. This amalgamation not only elevates the 4D features’ quality, rendering them more amenable for the bottleneck residual module to distill essential spatial and frequency insights, but also maintains the congruence of the input and output dimensions. In particular, the dimensions of the output data $X_{n}^{\prime }$, derived from the input 4D structural data $X_{n}$ within the discriminator, along with the dimensions of the output data $T_{n}^{\prime }$, which represent the outcome of the DeConv-processed data $T_{n}$ in the generator, are consistently preserved at $R^{h\times w\times d\times 2T}$. Through this approach, the model adeptly modulates and amplifies the spatial and frequency information’s weights within each time segment, ensuring the precision and salience of the data within the feature stream, and thereby significantly elevating the model’s overall performance.

#### 2.1.4. Bottleneck Residual Module

Standard Convolutional Neural Networks (CNNs) are extensively employed for their proficiency in extracting high-dimensional features pertinent to EEG signals within the realm of deep learning. Nonetheless, the advent of lightweight convolutions has garnered increasing attention amidst the burgeoning necessity for model deployment on low-power platforms characterized by constrained computational resources. This inclination is attributed to the compact architecture and enhanced efficiency of lightweight convolutions, positing them as a potential viable substitute for standard convolutions with expectations of broader application prospects in future endeavors. A pertinent study unveiled an efficient CNN variant, dubbed MobileNet, which endeavors to equilibrate the interplay among model size, computational speed, and accuracy, thereby furnishing an efficient solution for mobile and embedded systems [25]. MobileNet harness a streamlined design, employing Deep Separable Convolution (DSC) to forge lightweight deep neural networks. This approach empowers developers to tailor the network size appropriately, aligning it with the application’s resource limitations (e.g., latency, memory footprint). Empirical assessments across various scenarios have underscored MobileNet’s superiority in balancing resource consumption against accuracy. However, the ReLU activation function of DSC may lead to a large amount of missing feature information when processing lower dimensional outputs. In addition, training convolutional kernels during depthwise convolution (DC) is challenging, especially when a large number of convolutional kernel values tend to zero. Such phenomena not only diminish the model’s feature recognition capabilities, but may also instigate gradient vanishing issues, thereby impeding the learning efficacy and overall performance of the model.

**Figure 6:**
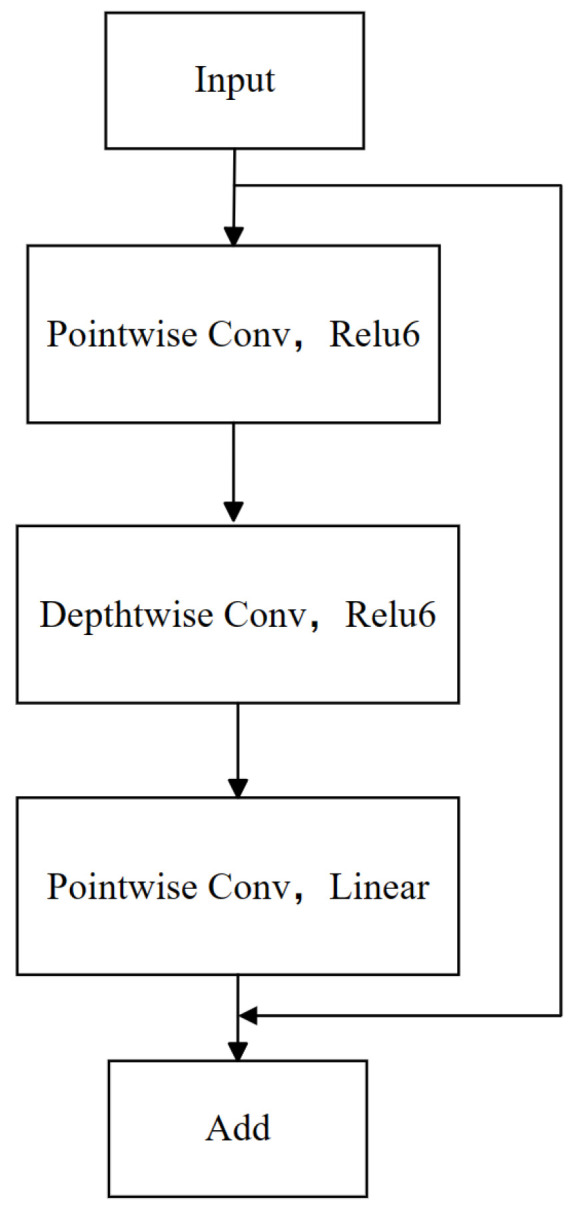
Bottleneck residual block.

(1) Bottleneck residual block (BR):

Inspired by the MobileNetV2 architecture [26], our model employs bottleneck residual blocks. Two core techniques were adopted to enhance the efficiency of feature extraction and reduce information loss. First, a reverse residual structure, characterized by diminished input–output dimensions and juxtaposed with an expanded middle layer dimension, is employed. This configuration facilitates the execution of convolutional operations in higher dimensions, thereby augmenting feature extraction capabilities and effectively curbing information loss during feature transfer. Subsequently, to further bolster the network’s efficiency in feature conduction, we introduced a linear bottleneck technique. This technique entails substituting the activation function in the network’s final layer from ReLU to linear activation. Such a modification preserves a greater extent of the original feature information, particularly within deep network structures, thus attenuating the potential for feature information loss attributable to nonlinear activation in low-dimensional outputs. Meanwhile, ReLU6 serves as the activation function in the other layers. As depicted in Figure 6, the bottleneck residual block integrated into our model comprises several core components: depthwise convolution (Depthwise Conv), pointwise convolution (Pointwise Conv), and the linear and ReLU6 activation functions.

The process enhances the network’s performance and stability. Initially, data are introduced into the network, followed by a stage of point convolution employing a 1×1×kd convolution kernel, where *k* dictates a multiplicative increase in the dimensionality of the middle layer within the bottleneck residual blocks to integrate input features. At this juncture, the ReLU6 activation function, a variation of the rectified linear unit (ReLU) that confines the output to a range between 0 and 6, is applied to introduce nonlinearity. Subsequently, a deep convolution operation, utilizing a 3×3×kd convolutional kernel and a stride of 1, is executed independently on each input channel, aiming to extract features in the spatial dimension while continuing to utilize the ReLU6 activation function to augment the nonlinear characteristics of the features. Following this, point convolution with a 1×1×C convolution kernel, where *C* specifies the dimensionality of the output feature layer, is employed once more, with a linear activation function, indicating that the output will directly reflect the input features without undergoing a nonlinear transformation. The culmination of this process involves integrating the output of the preceding point convolution and the original input through an addition operation, a technique termed residual join. This technique is specifically designed to mitigate the issue of gradient vanishing that deep learning networks might encounter during training sessions, thereby enhancing the stability of the training process. Collectively, this process amalgamates feature fusion, deep spatial feature extraction, and other strategies to augment the stability of network training.

EEG signals exhibit distinct characteristics from images, particularly regarding spatial and frequency correlations. This distinctive feature renders the bottleneck residual block particularly advantageous for the efficient extraction of spatial frequency features following the attention module.

(2) Bottleneck residual module

**Figure 7:**
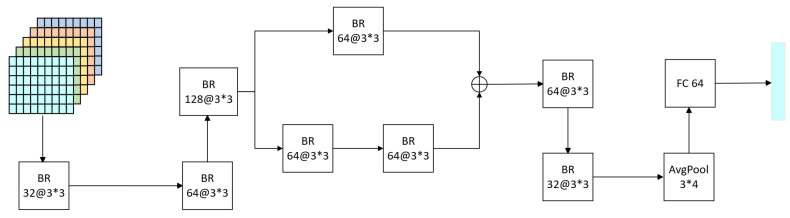
The bottleneck residual module in the discriminator, which has a slightly different structure in the generator.

The configurations of the bottleneck residual module exhibit minor variations in the architecture of both the generator and the discriminator; similarly, the bottleneck residual module includes eight bottleneck residual blocks and a two-branch structure, and the difference is that the bottleneck residual module in the discriminator has one more average pooling layer as well as one more fully-connected layer, as shown in Figure 7. When dealing with low-resolution feature maps such as EEG signals, we especially notice that although CNNs tend to reduce the size and computation of feature maps and prevent overfitting through multiple pooling layers, too much pooling may lead to the loss of important spatial information. To overcome this obstacle, a strategy involving the utilization of a solitary pooling layer has been adopted. This approach not only preserves the spatial integrity of the information, but also contributes to a reduction in the parameter count.

In the discriminator, the bottleneck residual module operates as follows: the input feature tensor $X_{n}^{\prime }$ is processed through a bottleneck residual convolution kernel of size 3×3. Initially, the feature map’s channel count is expanded from 5 to 128. Following a series of operations, this number is halved and subsequently directed into a bifurcated structure comprising two branches. Each branch houses a distinct arrangement of bottleneck residual blocks—one with a single block and the other with two—to amalgamate information across various receptive fields. The outputs from both branches are then combined, and the channel count is further reduced to 32 through the application of two bottleneck residual blocks. Subsequently, the feature map’s dimensions are condensed to 2×2 through a 3×4 averaging pooling layer. This layer not only diminishes the likelihood of overfitting, but also bolsters the network’s resilience. The resultant data from the pooling stage undergo expansion before being introduced into a fully connected layer comprising 64 nodes. This process yields an outcome, designated as $Q_{n}$, which exists within the realm of $Q_{n}\in R^{64\times 2T}$. It succinctly encapsulates both the spatial and frequency characteristics inherent to the segmented EEG signals initially obtained. These attributes are subsequently processed through the Transformer framework to distill temporal characteristics, thereby augmenting the model’s proficiency in identifying EEG signal characteristics. In the generator, the channel count is diminished to 5 through the concluding two bottleneck residual blocks. The feature tensor, $T_{n}^{\prime }$, processed by the attention module, serves as the input, culminating in the final output, $Y_{n}$, which represents the synthesized data produced by the generator.

#### 2.1.5. Transformer

**Figure 8:**
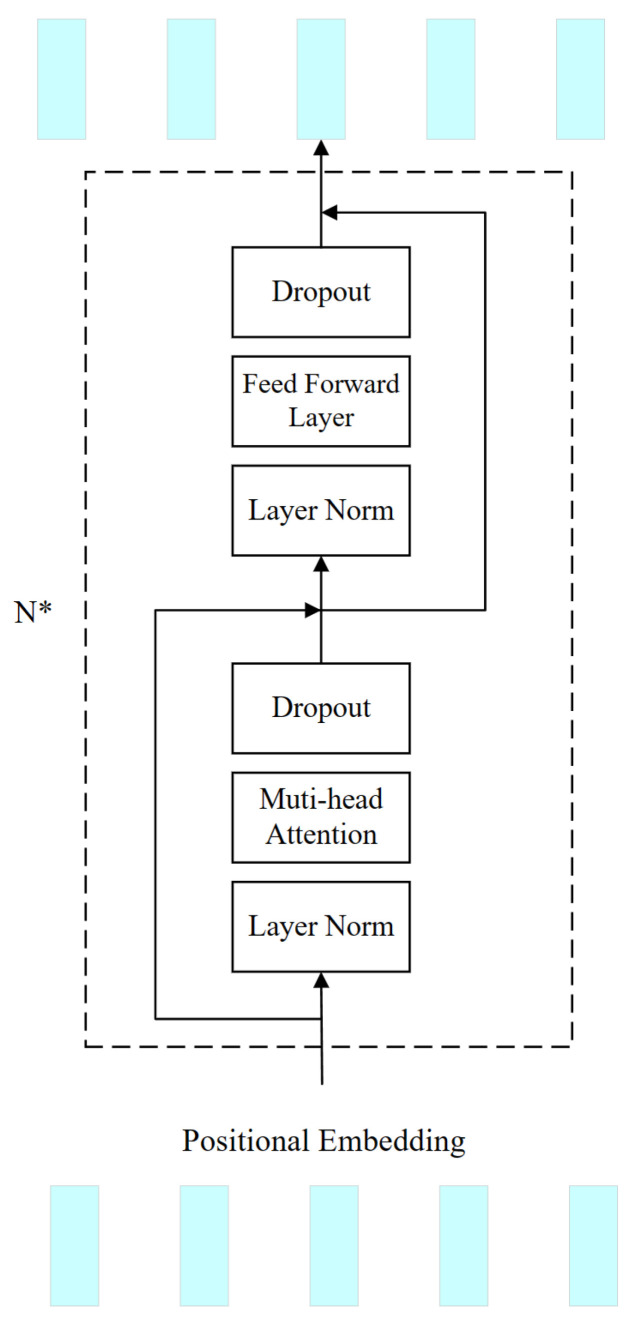
The Transformer module in the discriminator, and the module in the generator has the same structure. N* means repeating N times.

The architecture of the Transformer model is characterized by two principal elements: the multi-head self-attention mechanism and the feed-forward multilayer perceptron (MLP) segment, which employs a GELU as its activation function. To enhance the efficiency of the training process, a normalization layer is introduced between these two components. Furthermore, a dropout layer is incorporated subsequent to the output of each section to mitigate the risk of overfitting. Additionally, to maintain uninterrupted information flow and circumvent the challenges associated with vanishing or exploding gradients, a residual linking strategy is implemented within both segments. This approach is elucidated in Figure 8.

In our study, the Transformer modules of the generator and the discriminator are slightly different in terms of where they process the data stream. Specifically, within the discriminator, the Transformer module is utilized to discern temporal dependencies across time segments within the 4D features. Following the extraction of frequency and spatial characteristics from the 4D characteristic stream using a bottleneck residual module, the Transformer module commences temporal characteristic extraction, thus obtaining a comprehensive characterization of the EEG data in the dimensions of frequency, space, and time in an integrated manner. Upon the completion of EEG feature extraction, the derived features are subsequently input into the classification head to facilitate the categorization of fatigue status. In the generator, the Transformer module receives $w_{n}$ as its input and produces the output $w_{n}^{\prime }$, which subsequently serves as the input for the DeConv layer.

With the CA-ACGAN model, we are able to comprehensively extract the space, frequency and time characteristics of EEG data, which provides a powerful tool for fatigue detection.

### 2.2. Loss Function

In a CA-ACGAN, the loss functions employed by the generator *G* and the discriminator *D* incorporate category labeling, marking a substantial deviation from the loss functions utilized in traditional GANs. Specifically, see Equations (2) and (3):

(2) $$L_{D}=-L_{\mathrm{adv}}+\lambda L_{\mathrm{cls}}^{r}$$

(3) $$L_{G}=L_{\mathrm{adv}}+\lambda L_{\mathrm{cls}}^{f}$$

The adversarial loss $L_{\mathrm{adv}}$ quantifies the divergence between data synthesized by the generator and real data. Throughout the course of model training, the objective of generator *G* is to reduce the adversarial loss $L_{\mathrm{adv}}$, whereas the discriminator *D* endeavors to maximize it. The classification loss, $L_{\mathrm{cls}}$ assesses the discrepancy between the model-generated predictions and the actual classifications. Hyperparameter λ is used to adjust the significance of the categorization loss in the total loss function by varying the value of λ, and the relative importance between the categorization loss and the adversarial loss can be adjusted. In this investigation, λ was set to 1, signifying that the classification loss was attributed equal importance as the adversarial loss.

This loss function was meticulously crafted to enable the CA-ACGAN to not only fabricate data of high fidelity, but also to guarantee the precision of the generated data in categorical terms, thereby enhancing the model’s efficacy in specific tasks.

(1) Adversarial Loss:

In our investigation, we implemented an enhanced variant of Wasserstein’s generative adversarial network (WGAN) loss function, as delineated in reference [27]. The primary objective of the initial WGAN loss function centers on augmenting the fidelity of synthetically generated data by diminishing the Wasserstein distance between the distribution of the synthesized signals and that of the authentic signals. Nevertheless, the original WGAN necessitates the truncation of the discriminators’ weights during its execution, a requisite that could precipitate instability throughout the training regimen. To mitigate this issue, we introduced a gradient penalty term (GP), an innovative element within the loss function designed to penalize the magnitude of the gradient linked to the input and output of the discriminator, thereby ensuring a more stable training process.

The aforementioned loss function is formalized as in Equation (4):

(4) $$L_{\mathrm{adv}}=E_{x}\left[D_{\mathrm{adv}}\left(x\right)\right]-E_{\left(z,c\right)}\left[D_{\mathrm{adv}}\left(G\left(z,c\right)\right)\right]+\lambda_{\mathrm{gp}}E_{\hat{x}}\left[{\left({∥\nabla_{\hat{x}}D_{\mathrm{adv}}\left(\hat{x}\right)∥}_{2}-1\right)}^{2}\right]$$

In this formulation, $E_{x}\left[D_{\mathrm{adv}}\left(x\right)\right]$ signifies the expected output of the discriminator when presented with the real data, *x*, with the discriminator’s objective being to maximize this value to enhance its capability in accurately identifying real data. $E_{\left(z,c\right)}\left[D_{\mathrm{adv}}\left(G\left(z,c\right)\right)\right]$ depicts the anticipated outcome of the discriminator to the generator’s created data, which utilizes the noise vector *z* and the conditional variable *c*; here, the discriminator aims to minimize this value to effectively distinguish between generated and real data. The term $\lambda_{\mathrm{gp}}$ denotes the coefficient of the gradient penalty, serving as a mechanism to regulate the strength of the gradient penalty, and was assigned a value of 10 within the scope of this study. The notation $\hat{x}$ refers to a sample drawn from a hybrid distribution that combines the real data distribution with that of the generator. The expression $∥\nabla_{\hat{x}}D_{\mathrm{adv}}\left(\hat{x}\right){∥}_{2}-1$ represents the difference between the gradient paradigm of the discriminator at point $\hat{x}$ and 1, which is used as a penalty term to guarantee the discriminator’s adherence to the 1-Lipschitz prerequisite, i.e., the gradient paradigm does not exceed 1.

(2) Classification Loss:

To augment the discriminator *D*’s classification capability, an extra classification header was appended to it, and a corrective classification loss was incorporated during the discriminator and generator training phases. This specific classification loss aims to enhance the discriminator’s efficiency when processing genuine data signals. Within the context of the classification task, the cross-entropy loss function performs the role of a prevalent metric for gauging the divergence between the label distribution predicted by the model and the genuine label distribution. This loss function’s precise formulation is delineated as follows (Equation (5)):

(5) $$L_{\mathrm{cls}}^{r}=E_{\left(x,c^{\prime }\right)}\left[-\log D_{\mathrm{cls}}\left(c^{\prime }|x\right)\right]$$

The symbol $c^{\prime }$ represents the categorical label associated with the real data signal. Through the minimization of this specified loss function, the discriminator is honed to precisely categorize the real signal *x* into its authentic category $c^{\prime }$, thereby augmenting the model’s proficiency in accurately classifying real data.

In addition, the generator *G*’s objective is to ensure that the signal it generates can be accurately classified into a given category *c* by the discriminator. The adversarial loss expression is Equation (6):

(6) $$L_{\mathrm{cls}}^{f}=E_{\left(z,c\right)}\left[-\log D_{\mathrm{cls}}\left(c|G\left(z,c\right)\right)\right]$$

The expression $-\log D_{\mathrm{cls}}\left(c|G\left(z,c\right)\right)$ depicts the negative logarithm of the probability that the discriminator accurately classifies the signal G(z,c), generated by the generator, as belonging to category *c*, under the given condition *c*. Throughout the training process, the generator aims to minimize this loss value, thereby enhancing the conditional consistency of the data produced.

## 3. Experiments and Results

### 3.1. Experiments

#### 3.1.1. Dataset

We conducted an evaluation of our model utilizing the SEED-VIG dataset [28], a subset derived from the publicly accessible SEED dataset. In the following sections, we provide a comprehensive description of the SEED-VIG dataset and conclude the article with a link to access the dataset. Furthermore, we elucidate the methodology for preprocessing the SEED-VIG dataset, as well as the size of the SEED-VIG dataset, and specify how many categories it was classified into in our experiment.

The dataset known as SEED, made available through the collaborative efforts of the Brain-Inspired Computing Lab along with the Machine Intelligence Research Division at Shanghai Jiao Tong University, serves as an open-access resource for brainwave data. A subset of this project, SEED-VIG, focuses on the collection of brainwave and electrooculographic data specifically used for monitoring alertness levels.

To facilitate this research, an experimental setup was crafted to mimic a realistic four-lane highway driving scenario, displayed across a broad liquid crystal display monitor ahead of the participants. This simulation was designed to provide participants with real-time feedback on vehicle dynamics through interactions with a steering wheel and throttle, creating a driving experience that promotes driver fatigue by featuring a monotonous, predominantly straight roadway. The study engaged 23 participants and was strategically scheduled for the late afternoon, around 13:30, to coincide with the phase of the human circadian rhythm most susceptible to sleepiness. The entire experiment spanned approximately two hours, during which participants were asked to complete the driving simulation without any mechanisms designed to maintain alertness.

In this study, the Neuroscan apparatus was employed to concurrently capture EEG and EOG signals directly from the subjects’ foreheads, functioning at an elevated sampling frequency of 1000 Hz. The recording encompassed 12 electrodes positioned in the posterior brain regions (CP1, CPZ, CP2, P1, PZ, P2, PO3, POZ, PO4, O1, OZ, and O2) and 6 electrodes in the temporal areas (FT7, FT8, T7, T8, TP7, and TP8), all aligned based on the International 10–20 Electrode Placement System. An additional electrode, designated as CPZ, served as the reference electrode. Moreover, the individuals involved were equipped with SMI ETG spectacles designed for eye movement tracking to capture the dynamics of their gaze.

To evaluate the participants’ state of fatigue, this study employed the PERCLOS index, a metric derived from eye-tracking data. The PERCLOS index quantifies the degree of eye closure over a specified duration, calculated as the ratio of the duration of eye closure to the total observed period, as delineated in Equation (7):

(7) $$\mathrm{PERCLOS}=\frac{\mathrm{eye}\_\mathrm{closure}\_\mathrm{time}}{\mathrm{total}\_\mathrm{observed}\_\mathrm{time}}$$

The “total observed time” for the experimental procedure was established at 8 s, within which the duration of eye closure was meticulously recorded to ascertain the PERCLOS value, ranging from 0 to 1. PERCLOS values of 0.35 and 0.7 were used as the thresholds for judging wakefulness and fatigue and fatigue and drowsiness, respectively.

In our experiments, we removed a minute of data at the beginning and end of the experiment in order to exclude possible noise. It should be emphasized that we reduced the sampling rate of the original SEED-VIG dataset from 1000 Hz to 200 Hz. because based on the principles of the Nyquist–Shannon sampling theorem, it has been established that a sampling frequency of 200 Hz adequately encompasses the entirety of the information contained within the specified frequency regions. Only the EEG signals were included in this study and not the EOG signals. The data format of the processed EEG signal was 1,416,000 samples multiplied by 17 channels. Regarding EEG-based fatigue detection, the prevailing classification models predominantly utilized a binary classification scheme. Therefore, a threshold of 0.35 was established to differentiate between awake and fatigued states, thereby aligning with the benchmarks of existing fatigue detection frameworks for comparative analysis.

#### 3.1.2. Experimental Setup

In our deep learning laboratory, we provisioned leading-edge computational resources to facilitate the CA-ACGAN model’s optimal efficacy in detecting driver fatigue. These resources encompass GPU servers equipped with NVIDIA A100 Tensor Core GPUs, from Santa Clara, United States, real-time inference servers powered by dual Intel Xeon Scalable CPUs from Santa Clara, CA, USA, deep learning workstations integrated with NVIDIA RTX 3090 GPUs from Santa Clara, CA, USA, and data preprocessing servers configured with dual Intel Xeon Gold CPUs from Santa Clara, CA, USA.

For enhanced efficiency, we meticulously fine-tuned the hyperparameters of the CA-ACGAN model. Specifically, we adjusted the learning rates for the generator and discriminator to $1\times 10^{-4}$ and $3\times 10^{-4}$, respectively, and standardized the batch size for both components at 150. The model was trained utilizing the AdamW optimization algorithm, incorporating a 0.02 coefficient for weight decay. In tailoring the model’s loss function, we assigned values of 1 and 10 to λ and $\lambda_{\mathrm{gp}}$, respectively, to meet the specific requirements of the framework. Moreover, to validate the effectiveness of the framework, we implemented five-fold cross-validation and recorded the highest precision within the designated testing subset. To ensure the reproducibility of the experiments, we also fixed random seeds.

These hyperparameters were set based on our real-world, practical experiences with computational resources and general principles for training GAN models. While individualized parameter tuning for each case may have further improved the quality of the synthetic data, even with this generic configuration, our GAN model demonstrated higher effectiveness against other leading-edge models.

#### 3.1.3. Evaluation Metrics

(1) Classified metrics

For a comprehensive evaluation of the experimental outcomes, five widely recognized classification performance metrics were utilized: accuracy, F1 score, recall, precision, and the kappa coefficient. These metrics’ definitions are provided below:

- Accuracy: the proportion of all samples categorized accurately.
- Precision: the proportion of all samples that genuinely belong to the positive category out of all samples predicted as positive by the model.
- Recall: the proportion of true positives, indicating the fraction of all actual positive instances that the model successfully identifies.
- F1 score: defined as the weighted average between precision and recall, offering a comprehensive assessment of both metrics.
- Kappa coefficient: assesses the level of consistency in the classification performance, taking into account the possibility of chance agreement.

Together, these metrics provide us with a comprehensive evaluation framework for measuring the model performance on the task of classifying multi-category data from EEG signals.

(2) Generated data quality metric

The interpretation of time series data frequently poses challenges for human understanding due to the absence of standardized and uniform metrics with which to assess similarities between GAN-generated time series data and real data. An exhaustive literature review revealed that current methods for evaluating signal similarity fall short in distinguishing minor discrepancies between two signal sets effectively. Consequently, identifying a metric that exhibits high variability across different categories while maintaining low variability within the same category is crucial for the precise evaluation of similarities between real and synthesized signals. To meet this necessity, we employed a revised rendition of the wavelet coherence metric. This rendition enables a quantitative evaluation of the resemblance between two signal sets, eliminating the necessity to manually isolate features from the raw sequences [29].

The metric of wavelet coherence quantifies the synchronicity between two temporal sequences across the time-frequency spectrum, focusing on the consistency of phase interactions at distinct temporal points and frequency bands. This approach aims to uncover potential connections or causal relationships between them. The revised rendition of the wavelet coherence metric is derived by calculating the local correlation between the continuous wavelet transforms (CWT) of the two time series, as depicted in Equation (8):

(8) $$wc=\frac{{\left|S\left(\bar{C_{x}\left(a,b\right)}C_{y}\left(a,b\right)\right)\right|}^{2}}{S\left({\left|C_{x}\left(a,b\right)\right|}^{2}\right)\cdot S\left({\left|C_{y}\left(a,b\right)\right|}^{2}\right)}$$

In this formulation, the smoothing operator *S* enhances temporal and scale analysis, while the coefficients $\bar{C_{x}\left(a,b\right)}$ and $C_{y}\left(a,b\right)$ for signals *x* and *y* reflect the analysis’ focus on frequency (scale parameter *a*) and signal location (position parameter *b*), with the overline indicating complex conjugate operations. The *wc* value, ranging from 0 to 1, serves as an indicator of the coherence strength between the time series at the specified scale. Specifically, a higher value suggests enhanced coherence, implying a more consistent phase relationship within the time–frequency domain.

Wavelet coherence (*wc*), which quantifies the similarity between two time series signals, is represented as a two-dimensional matrix encompassing the dimensions of frequency and time step. To condense this matrix into a scalar value, we aggregated the values along the time axis (x-axis) and then calculated the mean across the frequency axis (y-axis), yielding a composite wavelet coherence score between the two signals. In the context of multidimensional signals, the average wavelet coherence across all dimensions was computed.

Further, for two groups of signals *A* and *B*, each containing *n* signal samples, we first calculated the wavelet coherence values between each signal in group *A* and then each signal in group *B*. Then, we summarized these values and calculated the average wavelet coherence value between the two groups of signals. This calculation process can be used to obtain a measure of the average similarity between the two groups of signals.

### 3.2. Result Analysis

#### 3.2.1. Classification Performance

A benchmarking analysis against four recognized network models was carried out to determine the relative effectiveness of the model introduced in this research: EEG Conv [30], EEG-Fest [31], LSTM-CapsAtt [32], and AMS-CNN [33].

In the dataset from SEED-VIG, the evaluation of various models was conducted using the five-fold cross-validation technique to calculate their mean performance metrics. The outcomes from the comparative study are documented in Table 1.

The CA-ACGAN model showcases highly precise fatigue detection capabilities through the utilization of EEG signals, outperforming other EEG-based models in terms of efficiency. The main challenge faced by the AMS-CNN is that it may demand significant quantities of data and computational power to adapt to different scales of feature extraction, which may result in extended periods of training and increased expenses related to computation. Furthermore, models such as EEG Conv exhibit limited adaptability to the complex and nonlinear characteristics of SEED-VIG dataset, hindering their performance in handling intricate EEG signal processing tasks. The EEG-Fest model incompletely utilizes the valuable information inherent in the SEED-VIG dataset, resulting in suboptimal outcomes during the processes of data analysis and interpretation. The LSTM-CapsAtt model requires further refinement to address challenges associated with noise interference and signal interpretation difficulties.

The distinctiveness of the CA-ACGAN lies in its incorporation of the bottleneck residual module, attention module, and Transformer module, which were crafted to efficiently extract and process spatial–frequency–temporal information. This capability not only ensures the model’s accuracy in the SEED-VIG dataset, but also its interpretability in EEG signal-based fatigue detection tasks, highlighting CA-ACGAN’s substantial benefits in managing complex EEG signal processing challenges.

**Figure 9:**
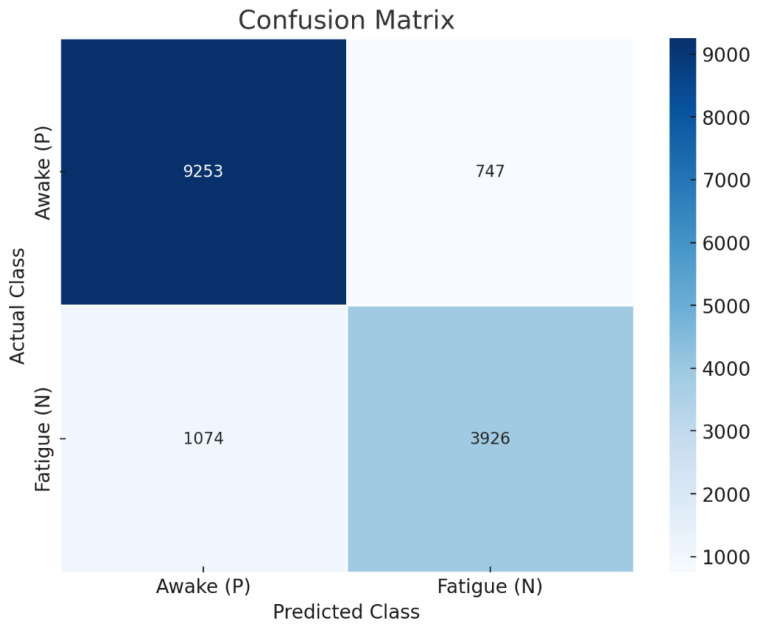
Confusion matrix for the CA-ACGAN model, demonstrating its classification performance on 15,000 imbalanced samples (10,000 awake, 5000 fatigue).

To evaluate the classification performance of the CA-ACGAN model under conditions of class imbalance, we conducted a test using a dataset comprising 15,000 randomly selected samples across two categories from 23 subjects, which included 10,000 samples labeled as ‘awake’ and 5000 as ‘fatigue’. The classification outcomes are presented in a confusion matrix (Figure 9). The results suggest that the proposed model maintains high accuracy in identifying categories, even when faced with an imbalanced dataset.

#### 3.2.2. Quality of Data Generated

In this research, wavelet coherence scores served as the metric for assessing the resemblance between synthesized signals that have been subjected to dimensionality reduction and their real counterparts, where a higher score denotes greater similarity. Furthermore, we compared the CA-ACGAN with the latest leading-edge time series GAN frameworks, including cDCGAN [34], CS-GAN [35], SigCWGAN [16], and GANSER [36]. Table 2 presents the results of the wavelet coherence analysis using the SEED-VIG dataset.

As shown by the data in Table 2, our CA-ACGAN model outperformed all other baseline models in terms of wavelet coherence scores. This superior performance suggests that the CA-ACGAN model is capable of synthesizing signals that bear a closer resemblance to real signals when compared to other GAN models. Our research integrates the strengths found in previous studies. We incorporated conditional data, akin to that used in a cDCGAN, to enhance the functionality of generative models and implement a Wasserstein loss, akin to that used in a SigCWGAN, to enhance the training process of our model. Similar to CS-GAN, our methodology also fully leverages the temporal and spatial attributes of EEG data. The GANSER model offers us valuable insights into the efficient utilization of amplified samples.

In this study, the classification model was trained using diverse training scenarios to evaluate its effectiveness in classification tasks. The training scenarios were varied and included the following: the utilization of purely real signal samples, the employment of purely synthetic signal samples, the application of a few real signal samples, and hybrid training incorporating a few real signal samples with mostly synthetic signal samples. The aggregate volume of data encompassed by the aforementioned four training scenarios remained equivalent. The outcomes of these experiments and the respective classification scores are detailed in Table 3.

The experimental findings may be condensed into the following points. 1. The synthetic signal, although not as effective as the purely real signal training, in terms of classification performance, can still provide similar information as the real signal. 2. A small dataset size poses challenges for the model’s learning capabilities. 3. A hybrid training approach, combining real and synthetic signals, surpasses the effectiveness of training with limited real signals or exclusively with synthetic signals. This approach mirrors a practical scenario where real signals are scarce, and synthetic signal support is beneficial.

In order to effectively combine real and synthetic signals to boost the model’s efficiency, we used a classification confidence-based selection method to filter high-quality generated signals [37]. This method involves assigning a confidence score to each synthetic signal point and establishing a threshold of 90 for this score. Only synthetic signal points that met or exceeded this threshold were selected. These selected, high-quality synthetic signal points, along with their categorical labels, were subsequently incorporated into the original training dataset, thereby augmenting the training set.

The adoption of a classification confidence-based strategy markedly improved the classification accuracy, elevating it to 0.938. It is crucial, however, to acknowledge that while this strategy significantly enhanced accuracy, it may also lead to increased computational time.

#### 3.2.3. Ablation Experiments

To assess the influence of the 4D feature flow, attention mechanisms, and lightweight convolution on the efficacy of the framework, we executed a comprehensive set of ablation experiments. These investigations contrast the fatigue detection capabilities of the comprehensive CA-ACGAN model against its numerous variants: CA-ACGAN (3D), which lacks 4D feature flow; CA-ACGAN equipped with sparse graphs; CA-ACGAN, devoid of the attention module; and CA-ACGAN, utilizing a conventional convolution module, as delineated in Table 4.

The results presented in Table 4 elucidate that the incorporation of 4D feature streams markedly augments model performance relative to the 3D features. Usually, there are two types of 2D images formed by electrodes: one is dense and the other is sparse. Empirical evidence from this study indicates that the dense image variant surpasses its sparse counterpart in both accuracy and training time efficiency. This discrepancy may stem from the inclusion of zeros between adjacent electrodes in sparse images, which fails to contribute substantive information. Conversely, the dense image type exhibits reduced training duration costs, potentially attributed to its diminished size, thereby lessening the requisite number of convolutional filters for processing. Although the integration of the attention module did not yield a substantial performance enhancement, it was designed to enhance the system’s proficiency in identifying key elements across the spatial and frequency domains and to reduce attention to unimportant features, which is beneficial to the overall efficacy of the model. Given the pronounced correlation between brainwave signals in both the spatial and frequency domains, the bottleneck residual block proves exceptionally efficacious for the extraction of spatial and frequency features. This model’s application not only minimizes the parameter count, but also significantly amplifies performance, underscoring its critical role in feature extraction.

## 4. Discussion and Future Perspectives

The comparative analysis presented herein elucidates that our proposed architecture surpasses alternative approaches in terms of the performance metrics. We further delve into the following noteworthy aspects:

(1) The integration of the dimensionality of the frequency, space, and time of the EEG signal data into a 4D feature dataset significantly enhances performance over 2D and 3D datasets, which encapsulate only fragmentary information. Employing this 4D feature dataset as input, our model achieved superior accuracy compared to existing methods, primarily due to its efficient structuring of input features.
(2) CNNs excel in local information extraction and are apt for spatial feature analysis, whereas Transformers excel in global information extraction and are suited for sequence data processing. The amalgamation of CNN and Transformer attributes lead to an improvement in model performance.

Despite the preliminary successes of this research, there exist multiple challenges and prospects for forthcoming studies that warrant exploration:

(1) The general applicability of the model: Subsequent research should examine the model’s applicability and efficiency across diverse EEG dataset types to further validate its widespread application potential.
(2) Real-time monitoring applications: The feasibility of incorporating the model into a real-time monitoring system should be explored, aiming to enhance service effectiveness in actual driving scenarios.
(3) Model optimization: Employing manual calculations for a preliminary estimate, alongside automated tools for a more thorough and accurate performance evaluation, we determined the computational complexity of the model to be approximately 0.21 GFLOPs. Future efforts should focus on identifying novel algorithms and techniques to diminish the model’s computational demands, thereby augmenting the operational efficiency and utility of the monitoring system.

## 5. Conclusions

In our research, we investigated an approach to detect driving fatigue through the utilization of the CA-ACGAN algorithm. This methodology adeptly identified patterns of brain activity by scrutinizing the four-dimensional characteristics of EEG data, which include frequency, spatial, and temporal attributes. Moreover, by integrating differential entropy (DE) features with electrode location data, we generated two-dimensional representations that elucidate spatial correlations, thereby aiming to augment the interpretation of signal intricacy.

Regarding the architectural composition of the model, the CA-ACGAN significantly enhanced model efficacy through the adoption of spatial and frequency attention mechanisms. This enabled the precise identification and processing of critical information within the EEG signals. The incorporation of a bottleneck residual module, along with depth and pointwise convolution techniques, facilitated the extraction of spatial and frequency features while maintaining model efficiency and compactness. The Transformer module was instrumental in capturing the temporal relationships of the four-dimensional features, improving feature detection capabilities through multi-head self-attention and a feed-forward multilayer perceptron (MLP).

During the experimental phase, the efficacy of the CA-ACGAN model was evaluated through hyperparameter optimization within a high-performance computing context, while employing various assessment metrics. The findings attest to the superior performance of the CA-ACGAN in detecting fatigue driving, particularly regarding the generative quality and classification accuracy. An ablation study further underscores the pivotal contributions of the four-dimensional feature mapping framework, the bottleneck residual module, and the compact 2D mapping to model enhancement.

In summary, the CA-ACGAN model exhibits considerable potential in applications related to fatigue driving detection. Integrating the multidimensional features of EEG signals with an innovative network architecture significantly enhances both the accuracy and practical value of fatigue recognition. This study distinguishes itself not only through the quality of the generated data, but also through its notable advancements in classification efficiency. Such achievements herald a new direction in the technology of fatigue driving detection, underscoring the model’s pivotal role in evolving the field.

## Figures and Tables

**Table 1:** Comparison of classification results.

Model	Accuracy	Recall	Precision	F1-Score	Kappa
**CA-ACGAN**	**0.907**	**0.954**	**0.916**	**0.935**	**0.814**
EEG Conv [30]	0.794	0.895	0.803	0.847	0.539
EEG-Fest [31]	0.821	0.882	0.814	0.847	0.528
LSTM-CapsAtt [32]	0.883	0.926	0.878	0.901	0.797
AMS-CNN [33]	0.803	0.879	0.821	0.849	0.556

**Table 2:** Wavelet coherence scores; higher scores are better.

Model	Score (%)
**CA-ACGAN**	**81.26**
cDCGAN [34]	69.83
CS-GAN [35]	76.49
SigCWGAN [16]	74.30
GANSER [36]	79.18

**Table 3:** Classification scores for training using different signal sources.

Data Mixing Methods	Accuracy
Purely real samples	**0.907**
Purely synthetic samples	0.864
Few real samples	0.805
Few real samples + most synthetic samples	**0.887**

**Table 4:** Results of ablation experiments.

Model	Accuracy	Recall	Precision	F1-Score	Kappa
**CA-ACGAN**	**0.907**	**0.954**	**0.916**	**0.935**	**0.814**
CA-ACGAN (3D)	0.872	0.926	0.881	0.903	0.787
CA-ACGAN (with sparse graphs)	0.893	0.943	0.904	0.923	0.798
CA-ACGAN (without attention module)	0.906	0.952	0.913	0.932	0.810
CA-ACGAN (with standard convolution module)	0.886	0.931	0.887	0.908	0.792

## Data Availability

Publicly available datasets were analyzed in this study. The data can be found here: https://bcmi.sjtu.edu.cn/home/seed/seed-vig.html (accessed on 20 April 2024).

## Abbreviations

*z*	noise vector
*l*	target categorical label
$W_{n}$	output of linear transformation in generator
$W_{n}^{\prime }$	$W_{n}$ after Transformer module in generator
$T_{n}$	$W_{n}^{\prime }$ after Deconv layer in generator
$T_{n}^{\prime }$	$T_{n}$ after attention module in generator
$Y_{n}$	synthetic data of 4D structure
$S_{n}$	raw EEG data
*m*	the number of electrode channels available
*r*	the rate of sampling
$D_{n}$	DE segment of 3D structure
*d*	the count of distinct frequency bands
*T*	EEG segments into time frames, amounting to 2T in total
*h*, *w*	the height and width of a 2D map
$X_{n}$	real data of a 4D structure
$X_{n}^{\prime }$	$X_{n}$ after attention module in discriminator
$Q_{n}$	$X_{n}^{\prime }$ after bottleneck residual module in discriminator
*C*	the dimensionality of the output layer within the bottleneck residual block
*k*	multiplicative dimensionality increase in bottleneck layer
